# Peer Review for “Administration Technique of Intranasal Corticosteroid Sprays Among Nepali Pharmacists: Cross-Sectional Study”

**DOI:** 10.2196/91443

**Published:** 2026-01-29

**Authors:** Ravi P Shankar

**Affiliations:** 1 Manipal College of Medical Sciences, Pokhara, Nepal

**Keywords:** intranasal corticosteroid spray, allergic rhinitis, device use technique, pharmacist, patient counselling, continuing pharmacy education

*This is a peer-review report for “Administration Technique of Intranasal Corticosteroid Sprays Among Nepali Pharmacists: Cross-Sectional Study.”*

## Round 1 Review

### General Comments

This is an important and well-written study [1]. My suggestions are listed below.

### Specific Comments

There are some problems with language and with unnecessary capitalization of words.

Page 3: INCS sprays should be defined in full on first mention in the text.

Page 8: Can details of the ethical committee that provided the approval be provided?

Was the informed consent obtained in writing?

Scoring system: Should the crucial steps not be provided with greater marks compared to the other steps?

Page 17: Please explain the classification tree (Chi-square automatic interaction detection method) for the benefit of the readers.

Page 17: “This research is one of a kind, conducted in Nepal.” Can this sentence be modified?

Page 19: Instead of continuing medical education (CME), continuing pharmacy education (CPE) may be a better term.

Page 20: What educational aids are you referring to?

Are the educational leaflets available in the Nepali language?

Page 20: “In our study, both the increasing age (>26 y old) were significantly associated with improved INCS [intranasal corticosteroid] counseling proficiency.” This sentence mentions both but then highlights only one factor.

Was this study conducted only in Kathmandu city and not in Lalitpur or Bhaktapur?

Page 21, Limitations section: Some of the findings may be extreme due to small subgroups or model overfitting. Can this be explained?

Different fonts are used in different locations, and this should be corrected.
